# The association of nonalcoholic fatty liver disease with central and peripheral blood pressure in adolescence: findings from a cross-sectional study

**DOI:** 10.1097/HJH.0000000000000445

**Published:** 2015-02-04

**Authors:** Sumaiya Patel, Debbie A. Lawlor, Diana L.S. Ferreira, Alun D. Hughes, Nish Chaturvedi, Mark Callaway, Chris Day, Naveed Sattar, Abigail Fraser

**Affiliations:** a School of Social and Community Medicine; b MRC Integrative Epidemiology Unit at the University of Bristol, Bristol; c Institute of Cardiovascular Science, University College London, London; d University Hospitals Bristol National Health Service Foundation Trust, Bristol; e Institute of Cellular Medicine (C.D.), Faculty of Medical Sciences, Newcastle University, Newcastle-upon-Tyne; f Institute of Cardiovascular & Medical Sciences, BHF Glasgow Cardiovascular Research Centre, Faculty of Medicine, University of Glasgow, Glasgow, UK.

**Keywords:** Avon Longitudinal Study of Parents and Children, blood pressure, children, nonalcoholic fatty liver disease, obesity

## Abstract

**Objectives:**

We aimed to determine the association of nonalcoholic fatty liver disease (NAFLD) with central and peripheral blood pressure (BP), in a general adolescent population and to examine whether associations are independent of adiposity.

**Methods:**

Using cross-sectional data from a subsample (*N* = 1904) of a UK birth cohort, we assessed markers of NAFLD including ultrasound scan (USS) determined fatty liver, shear velocity (marker of liver fibrosis), alanine aminotransferase (ALT), aspartate aminotransferase (AST) and gamma-glutamyltransferase (GGT) at a mean age of 17.8 years. These were related to BP [central and peripheral SBP and DBP and mean arterial pressure (MAP)].

**Results:**

Fatty liver was positively associated with central and peripheral SBP, DBP and MAP in models adjusting for age, sex, social class, puberty and alcohol intake. These positive associations were attenuated to the null when fat mass was included. For example, in confounder-adjusted models, not including fat mass, mean central SBP was 3.74 mmHg [95% confidence interval (CI) 1.12 to 6.36] higher in adolescents with USS fatty liver than in those without; with additional adjustment for fat mass, the association attenuated to the null value (−0.37 mmHg; 95% CI –3.09 to 2.36). Similar patterns were found for associations of ALT and GGT with central and peripheral BP. There was no consistent evidence of associations of shear velocity or AST with BP measurements. Fatty liver was not consistently associated with central pulse pressure (PP), peripheral PP and Aix@75.

**Conclusion:**

NAFLD is not associated with higher central or peripheral BP in adolescents once confounding by adiposity is taken into account.

## INTRODUCTION

Nonalcoholic fatty liver disease (NAFLD) is the accumulation of fat into the liver, in the absence of excess alcohol intake and other established causes [1]. NAFLD is associated with cardiovascular events [2,3] and cardiovascular disease (CVD) is the leading cause of death in people with NAFLD [2–4]. These observations have led to investigations into the potential mechanisms driving these association and there is now increasing evidence that NAFLD is associated with greater carotid intima-media thickness, a measure of atherosclerosis [5], endothelial dysfunction [6,7], dyslipidemia [8,9], as well as obesity, insulin resistance and diabetes [10], but the degree to which some of these associations are independent of other risk factors is not clear. In previous analyses using the data used here, we have shown that ultrasound scan (USS) determined NAFLD is associated with greater insulin resistance and dyslipidemia, even after adjustment for dual-energy X-ray absorptiometry (DEXA) determined total body fat mass [11].

Elevated blood pressure (BP) in adulthood is a strong determinant of cardiovascular risk [12,13]. BP tracks through the life course with BP in childhood or adolescence not only being associated with BP in adulthood [14,15] but also with adult CVD independently of BP in later life [16]. Peripheral (i.e. brachial) BP is the most common measurement taken, as it is easy to measure and a good predictor of cardiovascular risk. However, central BP is more strongly related to vascular damage [17] and may be a better predictor of cardiovascular events than peripheral BP [18]. Evidence also suggests that the difference between central BP and peripheral BP is more pronounced at younger ages [19].

Several studies in adults have found positive associations of NAFLD and higher liver enzymes with higher BP [9,10,20–23]. Studying these associations in younger populations is informative, as potential confounding factors such as smoking and use of medications affecting the liver, including antihypertensive agents, are much less prevalent than in adult populations.

The majority of studies examining the association of NAFLD with BP in paediatric or adolescent populations to date show that participants who are overweight/obese with NAFLD (diagnosed by either biopsy, MRI or USS) have higher BP than overweight/obese participants without NAFLD [24–28]. However, there is little evidence about these associations in the general adolescent population and it is unknown whether association differs for central BP compared with peripheral BP.

The aim of this study was to examine the association of USS-determined and blood-based measures of NAFLD and liver health with several central and peripheral BP measures [central and peripheral SBP, DBP, mean arterial pressure (MAP), central and peripheral pulse pressure (PP) and augmentation index] in adolescents and to determine whether any associations were explained by confounding due to adiposity, which is strongly associated with both NAFLD and BP.

## MATERIALS AND METHODS

### Study participants

The Avon Longitudinal Study of Parents and Children (ALSPAC) is a prospective, population-based birth cohort study that recruited 14 541 pregnancies, of which there were 13 867 live births from 13,761 women in Avon, UK, with expected dates of delivery 1 April 1991 to 31 December 1992 (http://www.alspac.bris.ac.uk) [29,30]. The cohort has been followed-up since birth with questionnaires and, from the age of 7, participants have been seen regularly in clinic; the most recent of these was the 17–18 year clinic assessment. This clinic assessment was attended by 5206 participants and included two separate substudies. In one, liver USS were conducted (*N* = 1935) and in the other, central and peripheral BP measurements were taken (*N* = 3896). To be eligible for the present study, participants had to have participated in the liver USS substudy. Singletons, and one randomly chosen twin from twin pairs, were included (*N* = 1917). In order to remove any effect of fat accumulation in the liver due to excess alcohol intake, consistent harmful alcohol drinkers were removed from the analysis. Information on participant's alcohol consumption was obtained using the Alcohol Use Disorders Identification Tests questionnaire [31]. This was administered at 16 years, and 17 years (at the same time as the USS assessment) and participants were scored between 0 and 20 with a score over 16 being classified as harmful alcohol consumption [31]. Consistent harmful alcohol drinkers were defined by a score of 16 or greater at both 16 and 17 years. After removal of 13 participants classified as consistent harmful drinkers, a sample of 1904 remained for the analysis.

Ethical approval for this study was obtained from the ALSPAC Law and Ethics Committee and the Local National Health Service Research Ethics Committee. All participants provided written informed consent.

### Liver ultrasound scans and blood-based outcomes

For both the liver USS and all blood-based analyses, participants were fasted overnight for those attending clinic in the morning, or for a minimum of 6 h, for those attending clinic after lunch.

### Liver ultrasound scans

A detailed description of the liver USS has been published previously [11]. Briefly, upper abdominal USS was completed by one of four trained sonographers using a Siemens Acuson S2000 USS system, with the participant at rest in the dorsal decubitus position. Echogenicity (a marker of liver fat) was assessed during deep inspiration and recorded as present, absent or uncertain according to established protocols using the right kidney as the reference organ [32].

Acoustic radiation force impulse-imaging (ARFI) of the right lobe of the liver was used to measure liver stiffness (or fibrosis), using standard protocols [33,34] and this was used as our main indicator of liver fibrosis. The right lobe of the liver was viewed through the intercostal space such that the pulse wave was traversing an area of at least 6 cm and was not traversing any major vascular structures and the right lobe was clearly viewed.

Shear velocity (in m/s) was assessed six times with a gap of at least 1 min between each measurement. The highest and lowest of these measurements were excluded and the Siemens Acuson S2000 system produced a mean of the remaining four measurements. If this mean was greater than 4 m/s, a further six measurements were taken from the left lobe. In the analyses, we have used the mean of four measurements after the highest and lowest velocities (of the six taken) were removed. When both right and left lobe values were available, the lowest mean of the two has been used.

### Assessment of blood-based measures

Fasting blood samples were immediately spun and frozen at –80^o^C. Measurements were assayed shortly (3–9 months) after samples were taken with no previous freeze-thaw cycles. All assays were completed in the same laboratory at the University of Glasgow. ALT, GGT and AST were measured by automated analyser with enzymatic methods.

### Assessment of central and peripheral blood pressure

Central BP and augmentation index was estimated using applanation tonometry with a SphygmoCor Px Pulse Wave Analysis System (Atcor Medical, NSW, Australia) at the radial artery. Recordings were calibrated using the brachial BP measured with the Omron 705 IT (Omron, The Netherlands) just prior to assessment of central pressure according to manufacturer instructions. Peripheral (brachial) BP was measured in triplicate, using a validated automated device (Omron 705 IT oscillometric BP monitor) using an appropriate size cuff, according to a standardized protocol. The average of the last two measurements was used in analyses. High SBP and DBP was defined using The Fourth Report on the Diagnosis, Evaluation, and Treatment of High Blood Pressure in Children and Adolescents [35]. MAP was calculated from the calibrated radial pressure waveforms. Augmentation pressure was calculated as the difference between the second and the first systolic shoulder of the central pressure wave curve, and the augmentation index was expressed as the percentage of augmentation from total PP. As the augmentation index is influenced in an inverse and linear manner by heart rate, augmentation index was normalized for a heart rate of 75 beats per minute (bpm) to give the augmentation index at 75 bpm (Aix@75). As it is unclear what the physiological meaning of negative values of augmentation index is [36], we also repeated the analysis with Aix@75, restricting the sample to those participants whose Aix@75 was at least 0.

### Assessment of other variables

Parental occupation was used to derive household occupational social class, with each household assigned the highest parental occupational [classes I (professional/managerial) to V (unskilled manual workers), using the 1991 British Office of Population and Census Statistics (OPCS) classification]. The participant's age was calculated in months from their date of birth and date of attendance at the clinic assessment. Offspring height was measured without shoes to the nearest 0.1 cm using a Harpenden stadiometer. A Lunar prodigy narrow fan beam densitometer was used to perform a whole body DEXA scan from which lean and fat mass were measured. Self-reported puberty stage, based on the Tanner staging system, was collected at age 17 by postal questionnaire. Pubertal stage was based on pubic hair staging for male participants and pubic hair and breast staging for female participants. If both were available, then the higher grade was used (taking the one suggesting higher stage if pubic hair and breast staging suggested different stages).

### Statistical analysis

All analysis was conducted using Stata version 12.0 MP2 (Stata Inc., College Station, Texas, USA). A series of multivariable regression models were constructed in order to examine the associations between measures of liver disease and BP and to explore the impact of adjustment for potential confounding factors. In the basic model (model 1), we controlled for age and sex. In the confounder-adjusted, minus fat mass, model (model 2), we additionally adjusted for household occupational social class, pubertal stage and alcohol intake. In the full confounder adjusted (model 3), we further adjusted for DEXA-assessed fat mass and included height and height-squared as covariables to remove any association of fat mass with height. In sensitivity analyses, we examined whether adjustment for BMI instead of DXA-assessed fat mass altered the associations examined (model 3).

### Dealing with missing data and additional analyses

Of the 1904 eligible participants (those included in the liver ultrasound study and not classed as consistent harmful drinkers), a proportion had missing data on any of exposures, outcomes and potential confounding factors [extent of missing for any single variable varied from 0 to 44.3% (Supplementary web table S1)]. To increase efficiency and minimize selection bias, we used multivariate multiple imputation to impute missing data for any of the eligible participants with missing data. We included all exposures, covariables, outcomes and potential predictors of missing data in the imputation. We generated 40 imputed datasets that were combined by Rubin's rules [37].

We repeated the multivariable regression analyses in complete cases (i.e. only including those participants who were eligible and had no missing data on exposures, covariables and outcomes) in order to compare the results with the imputed analyses.

## RESULTS

Table 1 summarizes the distribution of characteristics in the observed data for the eligible sample (i.e. those participants who had at least one USS measure and were not consistent hazardous drinkers). The prevalence of USS-determined fatty liver was 2.5%; no participants were classified as ‘uncertain’ with regard to the presence of fat in liver. The mean difference between central and peripheral SBP was 19.9 mmHg (SD = 4.8) Distributions of all variables were similar in the multivariate imputation databases and the observed data (see Supplementary web table S1).

Table 2 summarizes the multivariable associations of USS-determined fatty liver with measures of central and peripheral BP using the multivariate multiple imputation datasets. In the basic model (model 1), USS-determined fatty liver was positively associated with central and peripheral SBP, DBP and MAP. These associations attenuated slightly but persisted in the confounder, minus fat mass, adjusted model (model 2). Associations were attenuated to the null value when we additionally adjusted for confounding by fat mass (and height) (model 3). There was no strong evidence of associations of USS-determined fatty liver with central and peripheral PP in any of the models, with weak evidence of inverse associations upon adjustment for fat mass (model 3). USS-determined fatty liver was positively associated with augmentation index @75; however, the 95% confidence interval (95% CI) included the null value in all models.

Table 3 summarizes the multivariable associations of shear velocity, ALT, AST and GGT with measures of central and peripheral BP. Greater shear velocity (a marker of liver fibrosis) was associated with higher central and peripheral SBP in models 1 and 2, but associations were attenuated to the null value and coefficients became negative upon adjustment for fat mass (model 3). There was no strong evidence of associations with DBP, MAP and central and peripheral PP in any of the models.

Shear velocity was inversely associated with augmentation index @75 in all models. ALT and GGT were positively associated with all BP outcomes in the basic and confounder-adjusted models (models 1 and 2, respectively), although for augmentation index @75, the 95% CI spanned the null value. These positive associations were attenuated towards the null when fat mass was included in the model (model 3). There was no evidence of an association between AST and BP outcomes in any of the models.

Associations of USS-determined fatty liver, shear velocity and blood-based markers of NAFLD with augmentation index @75, restricted to those participants with values at least 0 (*N* = 852), are presented in Supplementary web table S2 for completion. Markers of NAFLD were generally inversely associated with augmentation index @75 at least 0, though CIs were wide due to the reduced sample size.

There were no notable differences in the associations of markers of NAFLD with BP outcomes when BMI was used as a measure of adiposity, compared with using DXA-assessed fat mass in model 3 (data not shown but available on request).

Supplementary web tables S3 and S4 show the multivariable associations of USS-determined fatty liver, and shear velocity and blood-based exposures, respectively, with measures of BP in a complete case analysis, that is only including those participants who were eligible and had no missing data on any exposures, outcomes and confounders (*N* = 438). Overall, results were similar to those presented here (using the multivariate multiple-imputed datasets), in terms of the direction of associations and how associations changed upon adjustment for fat mass, although the magnitude of the associations was larger, and CIs were wider due to the reduced sample size. The association between USS-determined NAFLD and MAP followed this same pattern, but the CI did not quite span the null value even when fully adjusted (model 3).

Supplementary web tables S5 and S6 show the multivariable associations of USS-determined fatty liver, and shear velocity and blood-based markers of NAFLD, respectively, with measures of high BP. Participants with USS-determined fatty liver, and with higher shear velocity, ALT, AST and GGT had greater odds of high SBP. This association was attenuated towards the null when adjusting for adiposity (model 3). Higher ALT and AST was associated with greater odds of high DBP and the same attenuation of the estimate was observed when adjusting for adiposity; however, the 95% CIs included the null value for all the models.

## DISCUSSION

In this study, we assessed liver health using USS to determine the presence of fat in the liver, ARFI to determine shear velocity (a marker of fibrosis) as well as measuring blood-based markers namely ALT, AST and GGT. There were no known cases of liver disease in this cohort and we removed the small number of participants who had reported consistent harmful drinking in the preceding 2 years. Therefore, it is reasonable to assume that in our cohort, USS-determined fatty liver is likely to represent NAFLD.

The aim of our study was firstly to examine the associations of NAFLD with central and peripheral BP and secondly to consider whether these associations are confounded by adiposity. The results demonstrate that for USS-determined NAFLD, ALT and GGT, there were positive associations with central and peripheral SBP and DBP and MAP. Interestingly, despite the large differences in central SBP and peripheral SBP, associations of NAFLD with both were of similar magnitude and CIs overlapped. We also found that positive associations were confounded by total body fat mass, with all attenuating towards the null value when adjusting for it. In contrast to the positive associations of USS-determined NAFLD with SBP and DBP in the main confounder-adjusted model, there were only weak associations with central PP and peripheral PP and Aix@75. Associations with central PP and peripheral PP became negative following adjustment for adiposity. PP depends on stroke volume, arterial stiffness and wave reflection [38]. In a study in this same cohort at a mean age of 10.6 years, overweight and obese participants had reduced arterial stiffness as measured by pulse wave velocity compared with normal weight participants [39] and a similar negative association between arterial stiffness and adiposity has been reported until in middle age [40]. This may explain the inverse association seen with PP when adjusting for adiposity.

Most previous studies that have reported the association of NAFLD with BP in children or adolescents have been in clinical populations who are overweight or obese [24–28]. Two studies in general (non-clinical) adolescents found higher BP in participants with USS-determined NAFLD than in those without NAFLD [41,42]. However, as the main endpoint of these studies was not BP, multivariable analysis was not conducted and hence whether this association was confounded by adiposity was not examined. Interestingly, using data from the Framingham Heart Study, Speliotes *et al.*[9] reported that adults with NAFLD (assessed using X-ray computed tomography) (*N* = 439) had higher SBP and a greater prevalence of hypertension than adults without NAFLD (*N* = 2150). However, as here, when adjusting for BMI, waist circumference and visceral adipose tissue (or adiposity traits), the association of NAFLD with SBP was attenuated, but the association with hypertension remained. Authors concluded that the association of NAFLD with BP may be due to confounding by adiposity.

In a separate publication based on this same cohort [11], we demonstrated that total and truncal fat mass are strongly associated with USS-determined NAFLD. We also found that associations of NAFLD with fasting glucose, insulin or adverse levels of lipids were somewhat but not fully attenuated upon adjustment for these adiposity measures (or BMI) [11]. By contrast, the results of our current study suggest that fat mass does fully confound the association of NAFLD with BP. These results suggest that the observed associations of NAFLD with CVD in adults may be due to NAFLD being a causal risk factor for adverse glucose, insulin and lipid concentrations and its effect on these, but not on BP. That said, it is still unclear whether NAFLD is a consequence and not a cause of insulin resistance, hyperglycaemia and dyslipidaemia. Alternatively, it is possible that any potential effect of NAFLD on BP is cumulative and will arise over time at a later age. As both the current study, and our previous study [11], were cross-sectional, we are unable to explore this further currently, though with further follow-up of this cohort, we hope to do so.

In our study, we have used several markers of liver health such as shear velocity (a marker of liver stiffness/fibrosis) and blood-based markers. Results were consistent across these, with the exception of AST that was not associated with BP in any of the models. This may be because AST is found in other organs such as muscle and as such is a less specific marker of liver fat levels than ALT and GGT [43,44].

### Strengths and limitations

The key strength of this study is that it is a large general adolescent population with several markers of NAFLD. Although we acknowledge that the number of participants with USS-determined NAFLD in our cohort is not large, we also have continuous markers of NAFLD that provide greater statistical power to detect associations and found that the results were generally consistent across these. We were able to consider both peripheral and central BP. The latter has been shown to be more strongly associated with CVD, and to our knowledge, the availability of central BP measures is unique to our study. USS-determined NAFLD is not the gold standard; however, liver biopsy would be neither ethical nor feasible to conduct in a large healthy population. Other methods such as MRI, although deemed a better imaging method, are much costlier and computed tomography (CT) scans are X-ray based and hence risk exposing a healthy young population to radiation. We were unable to adjust for physical activity or diet at age 17 years as a possible confounder, as these measures were unavailable. However, considering the attenuation of the associations seen when for adjusting for adiposity (i.e. the association is explained by confounding by adiposity), it is unlikely that including physical activity or dietary intake as confounders would have altered the interpretation of the results. Our study is cross-sectional, and therefore, we cannot assess causal effects or whether associations of NAFLD in adolescence with BP arise over time. The majority of this population are of European origin and we cannot assume that results generalize to other populations.

### Perspectives

In summary, in our cross-sectional analysis of a large adolescent cohort, markers of NAFLD were associated with greater central and peripheral BP, but these associations were fully confounded by, and therefore explained by, fat mass. These findings suggest that focusing on reducing general overweight/obesity in adolescents to help prevent future hypertension and its associated CVD risk is more important than a specific focus on NAFLD.

## ACKNOWLEDGEMENTS

We are extremely grateful to all of the families who took part in this study, the midwives for recruiting them and the whole ALSPAC team, which includes interviewers, computer and laboratory technicians, clerical workers, research scientists, volunteers, managers, receptionists and nurses.

The work presented in this article is supported by the British Heart Foundation (PG/11/33/28794), the European Union's Seventh Framework Programme (FP7/2007–2013) under grant agreement no. HEALTH-F2-2009-241762 for the project FLIP, both of which funded the salary of S.P. for her work on this project, and by the UK Medical Research Council (G0801456). The UK Medical Research Council and Wellcome Trust (092731) together with the University of Bristol provide core support for the ALSPAC study. Funding for peripheral and central pressure used in this publication came from grants from the Wellcome Trust (086676/7/08/Z) and British Heart Foundation (PG/06/145). D.A.L. and A.F. work in a Unit that receives funding from the UK Medical Research Council (MC_UU_12013/5). A.F. is funded by a UK Medical Research Council research fellowship (0701594).

### Conflicts of interest

There are no conflicts of interest.

## Supplementary Material

Supplemental Digital Content

## Figures and Tables

**TABLE 1:** Distribution of characteristics of the eligible sample (i.e. participants who attended the 17-year clinic and had at least one ultrasound scan measure)

Characteristic	*N*	Distribution mean (SD) for continuous variables or % for categorical variables
Male, % (*N*)	1904	41.5 (791)
Age (years)	1904	17.9 (0.4)
Manual social class, % (*N*)	1616	12.9 (209)
Postpuberty, % (*N*)	1059	82.7 (877)
Fat mass (kg), median (IQR)	1827	17.0 (11.4–24.0)
Truncal fat (g) median (IQR)	1819	8.4 (5.6–12.1)
BMI categories, % (*N*)	1847	
Underweight		8.0 (147)
Normal		68.0 (1255)
Overweight		17.0 (313)
Obese		7.0 (132)
Height (cm), mean (SD)	1847	170.7 (9.4)
Smoker, % (*N*)	1188	10.3 (122)
Alcohol intake (AUDIT score), % (*N*)	1704	
0–7		61.4 (1047)
8–15		33.2 (566)
16+		5.4 (92)
Heart rate (bpm), mean (SD)	1510	70.8 (10.8)
Ultrasound fatty liver, % (*N*)	1739	2.5 (43)
Shear velocity (m/s), median (IQR)	1742	1.2 (1.1–1.3)
ALT (U/l), median (IQR)	1293	15.7 (12.5–20.1)
AST(U/l), median (IQR)	1293	19.8 (17.0–23.4)
GGT (U/l), median (IQR)	1292	16.0 (13.0–21.0)
Central SBP (mmHg), mean (SD)	1512	96.0 (9.1)
Peripheral SBP (mmHg), mean (SD)	1512	115.8 (11.6)
High peripheral SBP (≥120 mmHg), % (*N*)^a^	1512	7.6
DBP (mmHg), mean (SD)	1512	63.5 (7.4)
High DBP (≥80 mmHg), % (*N*)^a^	1512	0.8
MAP (mmHg), mean (SD)	1512	79.7 (8.0)
Central pulse pressure (mmHg), mean (SD)	1512	31.2 (6.6)
Peripheral pulse pressure (mmHg), mean (SD)	1512	52.4 (10.4)
Augmentation index at heart rate 75 bpm (%), mean (SD)	1486	−1.5 (11.4)
Augmentation index at heart rate 75 bpm with values greater than 0 (%), mean (SD)	639	9.0 (6.6)

ALT, alanine aminotransferase; AST, aspartate aminotransferase; bpm, beats per minute; GGT, gamma-glutamyltransferase: IQR, interquartile range; SD, standard deviation: MAP, mean arterial pressur.

^a^Based on The Fourth Report on the Diagnosis, Evaluation and treatment of high Blood Pressure in Children and Adolescents [35].

**TABLE 2:** Multivariable associations [mean difference (95% confidence intervals)] of ultrasound scan determined fatty liver and with measures of central and peripheral blood pressure and arterial stiffness (*N* = 1904)

USS-determined fatty liver (yes vs. no)	Mean difference (95% confidence intervals)		
	Model 1	Model 2	Model 3
Central SBP (mmHg)	3.92 (1.32 to 6.52)	3.74 (1.12 to 6.36)	−0.37 (−3.09 to 2.36)
Peripheral SBP (mmHg)	4.00 (0.87 to 7.14)	3.82 (0.67 to 6.97)	−1.04 (−4.34 to 2.26)
DBP (mmHg)	4.00 (1.73 to 6.27)	3.91 (1.62 to 6.20)	1.05 (−1.33 to 3.42)
Mean arterial pressure (mmHg)	3.87 (1.42 to 6.32)	3.72 (1.24 to 6.20)	0.31 (−2.29 to 2.90)
Central pulse pressure (mmHg)	−0.33 (−2.16 to 1.51)	−0.41 (−2.24 to 1.42)	−1.74 (−3.67 to 0.18)
Peripheral pulse pressure (mmHg)	0.00 (−2.83 to 2.83)	−0.09 (−2.93 to 2.75)	−2.09 (−5.08 to 0.90)
Augmentation index at heart rate 75 bpm (%)	1.82 (−1.76 to 5.39)	1.69 (−1.91 to 5.30)	1.25 (−2.53 to 5.03)

Model 1 (basic model): adjusted for age at time of assessment and sex. Model 2 (confounder-adjusted model minus fat mass): as model 1 and additionally adjusted for social class, puberty and alcohol intake. Model 3 (adiposity-adjusted model): as model 2 and additionally adjusted for DEXA-assessed fat mass, height and height squared. bpm, beats per minute; USS, ultrasound scan.

**TABLE 3:** Multivariable associations [mean difference (95% confidence intervals)] of shear velocity and blood-based markers of nonalcoholic fatty liver disease with central and peripheral blood pressure measures (*N* = 1904)

	Mean difference (95% confidence intervals)		
	Model 1	Model 2	Model 3
Central SBP (mmHg)			
Shear velocity per SD (m/sec)	0.48 (0.06 to 0.90)	0.43 (0.01 to 0.85)	−0.16 (−0.58 to 0.27)
ALT per 10 U/l	0.90 (0.45 to 1.35)	0.84 (0.39 to 1.29)	0.27 (−0.19 to 0.73)
AST per 10 U/l	0.34 (−0.15 to 0.84)	0.34 (−0.16 to 0.85)	0.12 (−0.38 to 0.61)
GGT per 10 U/l	1.66 (1.08 to 2.25)	1.59 (1.00 to 2.18)	0.96 (0.34 to 1.59)
Peripheral SBP (mmHg)			
Shear velocity per SD (m/s)	0.63 (0.12 to 1.14)	0.59 (0.08 to 1.09)	−0.09 (−0.62 to 0.43)
ALT per 10 U/l	1.22 (0.68 to 1.76)	1.18 (0.64 to 1.73)	0.54 (−0.03 to 1.11)
AST per 10 U/l	0.51 (−0.11 to 1.13)	0.52 (−0.10 to 1.14)	0.26 (−0.35 to 0.86)
GGT per 10 U/l	1.99 (1.30 to 2.68)	1.96 (1.26 to 2.65)	1.24 (0.50 to 1.99)
DBP (mmHg)			
Shear velocity per SD (m/s)	0.25 (−0.11 to 0.60)	0.22 (−0.14 to 0.58)	−0.22 (−0.58 to 0.14)
ALT per 10 U/l	0.61 (0.22 to 1.00)	0.57 (0.18 to 0.97)	0.15 (−0.26 to 0.56)
AST per 10 U/l	0.06 (−0.40 to 0.51)	0.06 (−0.40 to 0.51)	−0.11 (−0.56 to 0.34)
GGT per 10 U/l	1.05 (0.55 to 1.55)	1.02 (0.51 to 1.52)	0.54 (0.00 to 1.07)
Mean arterial pressure (mmHg)			
Shear velocity per SD (m/s)	0.20 (−0.19 to 0.59)	0.16 (−0.23 to 0.55)	−0.37 (−0.76 to 0.02)
ALT per 10 U/l	0.75 (0.34 to 1.17)	0.70 (0.29 to 1.12)	0.20 (−0.22 to 0.63)
AST per 10 U/l	0.19 (−0.29 to 0.67)	0.20 (−0.28 to 0.68)	0.01 (−0.48 to 0.47)
GGT per 10 U/l	1.32 (0.79 to 1.85)	1.26 (0.72 to 1.80)	0.69 (0.13 to 1.25)
Central pulse pressure (mmHg)			
Shear velocity per SD (m/s)	0.27 (−0.03 to 0.57)	0.25 (−0.05 to 0.55)	0.10 (−0.22 to 0.41)
ALT per 10 U/l	0.33 (0.00 to 0.65)	0.31 (−0.01 to 0.62)	0.16 (−0.19 to 0.51)
AST per 10 U/l	0.29 (−0.08 to 0.67)	0.30 (−0.08 to 0.68)	0.24 (−0.14 to 0.62)
GGT per 10 U/l	0.62 (0.20 to 1.04)	0.58 (0.15 to 1.01)	0.43 (−0.04 to 0.90)
Peripheral pulse pressure (mmHg)			
Shear velocity per SD (m/s)	0.38 (−0.07 to 0.84)	0.37 (−0.09 to 0.82)	0.12 (−0.34 to 0.61)
ALT per 10 U/l	0.61 (0.12 to 1.10)	0.61 (0.11 to 1.10)	0.39 (−0.14 to 0.92)
AST per 10 U/l	0.45 (−0.10 to 1.00)	0.46 (−0.10 to 1.02)	0.37 (−0.19 to 0.93)
GGT per 10 U/l	0.94 (0.32 to 1.56)	0.94 (0.31 to 1.58)	0.71 (0.01 to 1.40)
Augmentation index at heart rate 75 bpm (%)			
Shear velocity per SD (m/s)	−0.52 (−1.08 to 0.04)	−0.57 (−1.13 to −0.02)	−0.73 (−1.31 to −0.15)
ALT per 10 U/l	0.24 (−0.42 to 0.90)	0.17 (−0.49 to 0.82)	0.05 (−0.65 to 0.75)
AST per 10 U/l	−0.09 (−0.82 to 0.63)	−0.10 (−0.82 to 0.62)	−0.16 (−0.89 to 0.56)
GGT per 10 U/l	0.88 (0.00 to 1.76)	0.75 (−0.15 to 1.64)	0.64 (−0.31 to 1.60)

Model 1 (basic model): adjusted for age at time of assessment and sex. Model 2 (confounder-adjusted model): as model 1 and additionally adjusted for social class, puberty and alcohol intake. Model 3 (adiposity-adjusted model): as model 2 and additionally adjusted for fat mass, height and height squared. ALT, alanine aminotransferase; AST, aspartate aminotransferase; GGT, gamma-glutamyltransferase.

## Reviewers’ Summary Evaluations

### Reviewer 1

The study suggests that the effect of non-alcoholic fatty liver disease (NAFLD) on blood pressure (BP) is only mediated by adiposity. The finding is interesting because other studies on the same issue, performed in populations of overweight and obese subjects, had obtained different results. A strength of the study is that in this case a population-based group of adolescents and young adults, participating in a prospective birth cohort study, was analysed. However, in the examined population the proportion of subjects with NAFLD was very low (2.5%) and this makes it quite difficult to investigate the association between NAFLD and BP. Moreover, it seems difficult to explain how, at the same obesity level, a subject with fatty liver can have the same metabolic conditions and the same cardiovascular risk as a subject without fatty liver. Other studies are necessary to better understand this important issue.

### Reviewer 2

The authors studied the relationship between NAFLD and several blood pressure-related measures. The strengths of this study are the use of ultrasound as well biochemical data to determine the presence of NAFLD, the analysis of a population-based sample, and the use of DEXA scans to thoroughly determine body composition. The prevalence of 2.5% NAFLD cases resulted in small number of subjects analyzed in group comparisons. This weak point is balanced by the generalizability of the findings. Overall, the association of NAFLD with blood pressure measures is completely dependent on body composition and annulled when this confounder is properly considered.
