# Supplementary material for: The association of nonalcoholic fatty liver disease with central and peripheral blood pressure in adolescence: findings from a cross-sectional study
Source: J Hypertens. 2015 Feb 4;33(3):546–53. doi: 10.1097/HJH.0000000000000445 (PMC4318641; doi:10.1097/HJH.0000000000000445)
Supplement: Supplemental Digital Content [file jhype-33-546-s001.docx]

**Web table S1: Distribution of characteristics in the imputed dataset (imputed up to those with at least one USS measure. N=1,904**

| **Variable** | **Number of participants in observed dataset** | **Percentage data missing** | **Distribution Mean (SE) or median (IQR) for continuous variables or % for categorical variables** | |
| --- | --- | --- | --- | --- |
|  |  |  | **Imputed dataset** | **Observed dataset** |
|  | **N** |  |  |  |
| Male (%) | 1,904 | 0 | 41.5 | 41.5 |
| Age (years), mean (SD) | 1,904 | 0 | 17.9 (0.01) | 17.9 (0.01) |
| Manual social class (%) | 1,616 | 15.1 | 16.8 | 12.9 |
| Post-puberty (%) | 1,059 | 44.3 | 81.8 | 82.7 |
| Fat mass (kg), median (IQR) | 1,827 | 4.0 | 16.9 (11.5, 23.9) | 17.0 (11.4, 24.0) |
| Truncal fat (kg), median (IQR) | 1,819 | 4.5 | 8.4 (5.6, 12.1) | 8.4 (5.6, 12.1) |
| Height (cm), mean (SD) | 1,847 | 3.0 | 170.7 (0.2) | 170.7 (0.2) |
| Smoker (%) | 1,187 | 37.7 | 12.5 | 10.3 |
| Alcohol intake (AUDIT score) (%)  0-7  8-15  16+ | 1,705 | 10.4 | 60.7  33.7  5.6 | 61.4  33.2  5.4 |
| Ultrasound fatty liver (%) | 1,738 | 8.7 | 2.7 | 2.5 |
| Shear velocity(m/sec) | 1,742 | 8.5 | 1.2 (1.1, 1.3) | 1.2 (1.1, 1.3) |
| ALT (U/l) | 1,293 | 32.1 | 15.8 (12.4, 20.6) | 15.7 (12.5, 20.1) |
| AST(U/l) | 1,293 | 32.1 | 19.9 (16.9, 23.9) | 19.8 (17.0, 23.4) |
| GGT (U/l) | 1,292 | 32.1 | 16.0 (13.0, 21.0) | 16.0 (13.0, 21.0) |
| Central systolic blood pressure (mmHg), mean (SD) | 1,512 | 20.6 | 96.0 (0.2) | 96.0 (0.2) |
| Peripheral systolic blood pressure (mmHg), mean (SD) | 1,512 | 20.6 | 115.8 (0.3) | 115.8 (0.3) |
| High peripheral systolic blood pressure, % (N) | 1,512 | 20.6 | 7.0 | 7.6 |
| Diastolic blood pressure (mmHg), mean (SD) | 1,512 | 20.6 | 63.5 (0.2) | 63.5 (0.2) |
| High diastolic blood pressure, % (N) | 1,512 | 20.6 | 0.7 | 0.8 |
| MAP (mmHg), mean (SD) | 1,512 | 20.6 | 79.7 (0.2) | 79.7 (0.2) |
| Central pulse pressure (mmHg), mean (SD) | 1,512 | 20.6 | 31.2 (0.2) | 31.2 (0.2) |
| Peripheral pulse pressure (mmHg), mean (SD) | 1,512 | 20.6 | 52.3 (0.3) | 52.4 (0.3) |
| Augmentation index @ heart rate 75bpm (%), mean (SD) | 1,486 | 22.0 | -1.5 (0.3) | -1.5 (0.3) |
| Augmentation index heart rate 75bpm (%) for those values ≥0^§^, mean (SD) | 639 | 20.5 ^§^ | 8.9 (0.2) | 9.0 (0.3) |

SE- standard error; ALT- alanine amino transferase; AST- aspartate amino transferase; GGT- gamma-glutamyltransferase; bpm: beats per minute

^§^ For augmentation index heart rate 75bpm (%) for those values ≥0

**Web table S2: Multivariable of markers of non-alcoholic fatty liver disease with augmentation index at 75 beats per minute (restricted to values** ≥**0, N=852)**

|  | **Mean difference (95% confidence intervals)** | | |
| --- | --- | --- | --- |
|  | **Model 1** | **Model 2** | **Model 3** |
| USS-determined fatty liver (yes vs. no) | -0.44 (-3.67, 2.79) | -0.57 (-3.84, 2.69) | -0.40 (-3.94, 3.14) |
| Shear velocity per SD (m/sec) | 0.08 (-0.39, 0.56) | -0.03 (-0.45, 0.50) | 0.06 (-0.44, 0.57) |
| ALT per 10 U/l | -0.26 (-0.78, 0.25) | -0.30 (-0.82, 0.21) | -0.29 (-0.84, 0.25) |
| AST per 10 U/l | -0.26 (-1.04, 0.51) | -0.26 (-1.04, 0.51) | -0.26 (-1.04, 0.52) |
| GGT per 10 U/l | -0.06 (-0.65, 0.53) | -0.16 (-0.76, 0.44) | -0.12 (-0.77, 0.53) |

USS- ultrasound scan; ALT- alanine amino transferase; AST- aspartate amino transferase; GGT- gamma-glutamyl transferase

Model 1 (basic model): adjusted for age at time of assessment and gender

Model 2 (confounder adjusted model, minus fat mass): as model 1 plus additionally adjusted for social class, puberty and alcohol intake

Model 3 (adiposity adjusted model): as model 2 plus additionally adjusted for fat mass, height and height squared

**Web table S3: Complete case multivariable associations [mean difference (95% confidence intervals)] of ultrasound fatty liver with central and peripheral blood pressure measures N=438**

| **USS- determined fatty liver (yes vs. no)** | **Mean difference (95% confidence intervals)** | | |
| --- | --- | --- | --- |
|  | **Model 1** | **Model 2** | **Model 3** |
| Central systolic blood pressure (mmHg) | 10.92 (3.96, 17.87) | 11.28 (4.36, 18.20) | 6.15 (-0.73, 13.03) |
| Peripheral systolic blood pressure (mmHg) | 12.37 (4.00, 20.75) | 12.67 (4.29, 21.06) | 6.64 (-1.70, 14.98) |
| Diastolic blood pressure (mmHg) | 9.58 (3.56, 15.60) | 9.80 (3.77, 15.82) | 6.05 (-0.02, 12.11) |
| Mean arterial pressure (mmHg) | 10.08 (3.59, 16.58) | 10.41 (3.93, 16.89) | 7.29 (0.20, 14.38) |
| Central pulse pressure (mmHg) | 0.44 (-3.99, 4.86) | 0.56 (-3.88, 4.99) | -0.99 (-5.55, 3.57) |
| Peripheral pulse pressure (mmHg) | 2.80 (-4.17, 9.76) | 2.87 (-4.12, 9.87) | 0.60 (-6.60, 7.79) |
| Augmentation index at heart rate 75 beats per minute (%) | 1.87 (-7.98, 11.75) | 2.04 (7.80, 11.88) | 2.59 (-7.58, 12.77) |

USS-ultrasound scan

Model 1 (basic model): adjusted for age at time of assessment and gender

Model 2 (confounder adjusted mode, minus fat mass): as model 1 plus additionally adjusted for social class, puberty and alcohol intake

Model 3 (adiposity adjusted model): as model 2 plus additionally adjusted for fat mass, height and height squared

**Web table S4: Complete case multivariable associations of blood-based markers of non-alcoholic fatty liver disease with central and peripheral blood pressure measures N=438**

|  | **Mean difference (95% confidence interval)** | | |
| --- | --- | --- | --- |
|  | **Model 1** | **Model 2** | **Model 3** |
| **Central systolic blood pressure (mmHg)** | | | |
| Shear velocity per SD (m/sec) | 0.35 (-0.60, 1.29) | 0.31 (-0.63, 1.25) | -0.16 (-1.08, 0.75) |
| ALT per 10 U/l | 0.80 (0.13, 1.47) | 0.79 (0.12, 1.47) | 0.35 (-0.31, 1.02) |
| AST per 10 U/l | 0.22 (-0.84, 1.33) | 0.26 (-0.82, 1.35) | 0.15 (-0.89, 1.18) |
| GGT per 10 U/l | 2.06 (1.06, 3.05) | 1.93 (0.93, 2.93) | 1.22 (0.22, 2.23) |
| **Peripheral systolic blood pressure (mmHg)** | | | |
| Shear velocity per SD (m/sec) | 0.75 (-0.38, 1.88) | 0.73 (-0.40, 1.87) | 0.22 (-0.89, 1.32) |
| ALT per 10 U/l | 1.04 (0.24, 1.83) | 1.11 (0.30, 1.93) | 0.62 (-0.18, 1.43) |
| AST per 10 U/l | 0.52 (-0.78, 1.82) | 0.60 (-0.71, 1.91) | 0.47 (-0.78, 1.72) |
| GGT per 10 U/l | 2.58 (1.39, 3.78) | 2.57 (1.35, 3.78) | 1.77 (0.56, 2.98) |
| **Diastolic blood pressure (mmHg)** | | | |
| Shear velocity per SD (m/sec) | 0.10 (-0.72, 0.92) | 0.07 (-0.75, 0.88) | -0.29 (-1.10, 0.51) |
| ALT per 10 U/l | 0.66 (0.08, 1.23) | 0.66 (0.07, 1.24) | 0.32 (-0.27, 0.91) |
| AST per 10 U/l | -0.05 (-0.99, 0.88) | -0.04 (-0.98, 0.90) | -0.13 (-1.04, 0.78) |
| GGT per 10 U/l | 1.47 (0.60, 2.34) | 1.40 (0.52, 2.28) | 0.86 (-0.03, 1.74) |
| **Mean arterial pressure (mmHg)** | | | |
| Shear velocity per SD (m/sec) | -0.08 (-0.96, 0.80) | -0.12 (-1.00, 0.76) | -0.56 (-1.42, 0.30) |
| ALT per 10 U/l | 0.75 (0.13, 1.37) | 0.74 (0.11, 1.37) | 0.34 (-0.29, 0.97) |
| AST per 10 U/l | 0.16 (-0.84, 1.17) | 0.18 (-0.83, 1.18) | 0.06 (-0.91, 1.04) |
| GGT per 10 U/l | 1.76 (0.83, 2.70) | 1.66 (0.71, 2.60) | 1.03 (0.08, 1.98) |
| ***Arterial stiffness*** | | | |
| Shear velocity per SD (m/sec) | 0.34 (-0.26, 0.93) | 0.33 (-0.27, 0.92) | 0.22 (-0.38, 0.82) |
| ALT per 10 U/l | 0.18 (-0.24, 0.60) | 0.18 (-0.25, 0.60) | 0.07 (-0.37, 0.51) |
| AST per 10 U/l | 0.25 (-0.43, 0.93) | 0.25 (-0.43, 0.94) | 0.23 (-0.45, 0.91) |
| GGT per 10 U/l | 0.57 (-0.07, 1.21) | 0.52 (-0.12, 1.17) | 0.35 (-0.32, 1.01) |
| **Peripheral pulse pressure (mmHg)** | | | |
| Shear velocity per SD (m/sec) | 0.65 (-0.28, 1.58) | 0.66 (-0.28, 1.60) | 0.51 (-0.44, 1.46) |
| ALT per 10 U/l | 0.40 (-0.28, 1.04) | 0.46 (-0.22, 1.13) | 0.30 (-0.39, 0.99) |
| AST per 10 U/l | 0.57 (-0.50, 1.64) | 0.64 (-0.44, 1.72) | 0.60 (-0.47, 1.68) |
| GGT per 10 U/l | 1.11 (0.11, 2.12) | 1.16 (0.15, 2.18) | 0.91 (-0.13, 1.96) |
| **Augmentation index at heart rate 75 bpm (%)** | | | |
| Shear velocity per SD (m/sec) | -2.14 (-3.45, -0.83) | -2.20 (-3.51, -0.90) | -2.34 (-3.67, -1.01) |
| ALT per 10 U/l | -0.06 (-1.00, 0.88) | -0.26 (-1.21, 0.70) | -0.28 (-1.26, 0.70) |
| AST per 10 U/l | -0.24 (-1.76, 1.28) | -0.40 (-1.92, 1.12) | -0.43 (-1.95, 1.09) |
| GGT per 10 U/l | -0.02 (-1.45, 1.40) | -0.31 (-1.75, 1.13) | 0.32 (-1.80, 1.17) |

ALT- alanine amino transferase; AST- aspartate amino transferase; GGT- gamma-glutamyl transferase; bpm- beats per minute

Model 1 (basic model): adjusted for age at time of assessment and gender

Model 2 (confounder adjusted model, minus fat mass): as model 1 plus additionally adjusted for social class, puberty and alcohol intake

Model 3 (adiposity adjusted model): as model 2 plus additionally adjusted for fat mass, height and height squared

**Web table S5: Multivariable associations of USS-determined fatty liver and with measures of high blood pressure (N=1,904)**

| **USS- determined fatty liver (yes vs. no)** | **Odds ratio (95% confidence intervals)** | | |
| --- | --- | --- | --- |
|  | **Model 1** | **Model 2** | **Model 3** |
| High peripheral systolic blood pressure | 2.83 (1.25, 6.41) | 2.72 (1.18, 6.27) | 1.00 (0.38, 2.63) |
| High diastolic blood pressure | 6.84 (1.45, 32.39) | 6.25 (1.27, 30.79) | 3.72 (0.54, 25.88) |

USS- ultrasound scan; bpm- beats per minute

Model 1 (basic model): adjusted for age at time of assessment and gender

Model 2 (confounder adjusted model- minus fat mass): as model 1 plus additionally adjusted for social class, puberty and alcohol intake

Model 3 (adiposity adjusted model): as model 2 plus additionally adjusted for DEXA-assessed fat mass, height and height squared

**Web table S6: Multivariable associations of shear velocity and blood-based markers of non-alcoholic fatty liver disease with high blood pressure (N=1,904)**

|  | **Odds ratio (95% confidence intervals)** | | |
| --- | --- | --- | --- |
|  | **Model 1** | **Model 2** | **Model 3** |
| **High peripheral systolic blood pressure** | | | |
| Shear velocity per SD (m/sec) | 1.12 (0.92, 1.34) | 1.10 (0.92, 1.33) | 0.93 (0.76, 1.13) |
| ALT per 10 U/l | 1.31 (1.15, 1.49) | 1.31 (1.15, 1.50) | 1.16 (0.99, 1.35) |
| AST per 10 U/l | 1.12 (0.95, 1.31) | 1.12 (0.95, 1.32) | 1.06 (0.87, 1.28) |
| GGT per 10 U/l | 1.70 (1.41, 2.07) | 1.72 (1.41, 2.09) | 1.50 (1.21, 1.86) |
| **High diastolic blood pressure** | | | |
| Shear velocity per SD (m/sec) | 0.97 (0.53, 1.75) | 0.95 (0.52, 1.75) | 0.82 (0.46, 1.47) |
| ALT per 10 U/l | 1.21 (0.82, 1.80) | 1.19 (0.81, 1.76) | 1.06 (0.67, 1.69) |
| AST per 10 U/l | 1.01 (0.50, 2.03) | 1.00 (0.51, 2.00) | 0.92 (0.41, 2.08) |
| GGT per 10 U/l | 0.97 (0.39, 2.41) | 1.00 (0.41, 2.45) | 0.79 (0.28, 2.24) |

ALT- alanine amino transferase; AST- aspartate amino transferase; GGT- gamma-glutamyl transferase

Model 1 (basic model): adjusted for age at time of assessment and gender

Model 2 (confounder adjusted model): as model 1 plus additionally adjusted for social class, puberty and alcohol intake

Model 3 (adiposity adjusted model): as model 2 plus additionally adjusted for fat mass, height and height squared
